# Patient-reported outcome measures for retinoblastoma: a scoping review

**DOI:** 10.1186/s41687-020-00232-7

**Published:** 2020-08-08

**Authors:** Ana Janic, Sylvie Bowden, Sarah Levy, Jennifer Stinson, Helen Dimaras

**Affiliations:** 1 Faculty of Medicine, University of Toronto, 1 King’s College Circle, Toronto, M5S 1A8 Canada; 2 Department of Ophthalmology & Vision Sciences, The Hospital for Sick Children, 555 University Ave, Toronto, M5G 2R3 Canada; 3 Child Health Evaluative Sciences Program, SickKids Research Institute, 686 Bay Street, Toronto, M5G 0A4 Canada; 4 Department of Anesthesia and Pain Medicine, The Hospital for Sick Children, 555 University Ave, Toronto, M5G 2R3 Canada; 5 Lawrence S. Bloomberg Faculty of Nursing, University of Toronto, 155 College Street, Toronto, M5T 1P8 Canada; 6 The Centre for Global Child Health, SickKids Research Institute, 686 Bay Street, Toronto, M5G 0A4 Canada; 7 Division of Clinical Public Health, University of Toronto, 150 College Street, Toronto, M5T 3M7 Canada

**Keywords:** Patient-reported outcome measures, Retinoblastoma, Pediatric ophthalmology, Pediatric oncology, Measurement properties, Quality assessment

## Abstract

**Background:**

Retinoblastoma is a childhood retinal cancer with lifelong consequences such as vision loss and increased risk of second cancer. Patient-reported outcome measures (PROMs) are instruments that measure outcomes related to health directly reported by patients. The purpose of this study was to determine the scope, characteristics and quality of PROMs used in retinoblastoma and related fields of pediatric ophthalmology and pediatric oncology.

**Methods:**

Databases MEDLINE and Embase were searched for studies in the English language that reported on PROMs used in retinoblastoma, pediatric oncology, or pediatric ophthalmology; grey literature and studies reporting on developmental PROM phases were excluded. PROMs were grouped by the construct measured and domains assessed, and classified as condition-specific or generic. A subsequent search was then conducted in MEDLINE and Embase for studies assessing measurement properties of the identified PROMs. PROMs with associated studies were assessed for their methodologic quality using the COnsensus-based standard for the Selection of health Measurement INstruments (COSMIN) strategy.

**Results:**

Among 110 eligible studies uncovered by the database searches, 143 PROMs were identified: one retinoblastoma-specific, 56 ophthalmology- and 86 oncology-related. The most common construct measured was ‘health-related quality of life’ and the most common domain assessed was emotional well-being. Of the 143 PROMs, 100 had associated validation studies; the one retinoblastoma-specific PROM was not validated. Quality assessment revealed 34/100 PROMs received a score of sufficient quality in both subcategories of ‘overall content validity’; 3/100 received a score of sufficient quality in both subcategories of ‘internal structure’; 0/100 received a score of sufficient quality in all three subcategories of ‘remaining measurement properties’. The Patient-Reported Outcome Measure Information System (PROMIS) Pediatric Profile-25 was the highest-scoring PROM identified, meeting COSMIN standards for 2/3 measurement property categories (and 5/7 subcategories). Eleven additional PROMs were identified which had sufficient scores in 1/3 measurement property categories (and 5/7 subcategories).

**Conclusion:**

The study identified several PROMs from the pediatric ophthalmology and pediatric oncology literature that could be relevant to the retinoblastoma population, but many have limits to their validation. Future development of a retinoblastoma-specific PROM, performed in partnership with retinoblastoma patients to support optimal content validity, could first focus on the selection and definition of the optimal construct to measure, followed potentially by adaptation and further validation of the relevant PROMs with strong methodologic quality identified in this study.

## Introduction

Retinoblastoma is a rare, intraocular cancer that arises in the developing retina, primarily due to bi-allelic inactivation of the *RB1* gene [1, 2]. With uniform global incidence, approximately 8000 children are diagnosed with the disease annually [1]. In high-income countries, the retinoblastoma survival rate is 96%, yet the cancer carries lifelong consequences that can affect vision and quality of life [3].

Treatment options for patients diagnosed with retinoblastoma vary depending on tumor size and location [3]. Smaller tumors located away from the center of vision are generally treated with focal laser therapy, while larger tumors could warrant enucleation (surgery to remove the eye), or more aggressive therapies to save the eye, like chemotherapy, or external beam radiation therapy [1, 3]. Treatment decisions also depend on whether or not tumor is present in one (unilateral) or both (bilateral) eyes [1, 3]. Unsurprisingly then, long-term clinical outcomes can differ among patients based on their initial diagnosis and treatment [3–6].

In addition to clinical outcomes, there are other consequences for patients that can impact a retinoblastoma survivor. For example, research indicates that survivors who have undergone enucleation or radiation therapy experience distress regarding their cosmetic appearance [3, 7, 8]. Having to wear a prosthetic eye or live with cranial-facial deformities induced by radiation, can foster self-consciousness and induce social anxiety [1, 3]. Furthermore, approximately half of survivors carry a constitutional *RB1* mutation (heritable retinoblastoma) [1, 2], which confers an increased risk of second malignancies and potential to pass the disease causing allele to future offspring [1, 3, 4]. Survivors and their caregivers have indicated that the risks associated with heritable retinoblastoma are a source of worry and anxiety [4–6, 9].

There is an increasing emphasis on patient-focused evaluation of disease impact [10–12]. This has led to the creation of instruments intended to capture the subjective assessment of health outcomes from the patient’s perspective. Known as patient-reported outcome measures (PROMs), these instruments capture a variety of health-related outcomes and are directly reported on by patients themselves [13–15]. PROMs can be administered via hard copy or electronic self-report questionnaires, as well as through in-person or telephone interviews [13–15].

Over the past 50 years, PROMs have been used in clinical trials [16–18], national audits [19, 20], and registers for condition-specific databases [21]. During the past two decades, widespread interest in using PROMs to track and monitor outcomes in clinical practice has further increased [12, 13]. PROMs can facilitate open communication between patients, families, and health care professionals [12, 18]. They provide unique information on the impact of a medical condition and its treatment from the patients’ perspective [13, 18, 22], and can be used for continual monitoring of patient progress, offering the possibility of early detection of secondary problems [18, 22, 23]. Further, PROMs can provide researchers and clinicians with valuable data that can catalyze more focused research, answer currently pending research questions, or inform clinical decision making [22].

It follows then, that a retinoblastoma-specific PROM could be relevant for a population with such significant and varied outcomes over their life course. There are a number of PROMs used in pediatric oncology and pediatric ophthalmology [24–27], yet it is unclear if (i) these are used or relevant for retinoblastoma or (ii) condition-specific measures for retinoblastoma exist. Before developing a retinoblastoma-specific PROM, it is important to first identify PROMs that might be relevant for retinoblastoma patients. It is equally important to assess which outcomes are evaluated by these PROMs and if the PROMs are methodologically sound – that is, which PROMs measure what is meant to be measured accurately (validity) and do they measure what is meant to be measured consistently (reliability).

## Methods

### Primary objective

To identify and characterize PROMs currently used for retinoblastoma, pediatric ophthalmology and pediatric oncology research.

#### Search strategy

A search strategy was developed with the assistance of an information scientist. The search was conducted in databases MEDLINE and Embase. These databases were posited to be most relevant to the study aims due to their focus on biomedical life science and therefore ensured comprehensive capture of articles. Search terms included “Patient-Reported Outcome” AND “Patient-Reported Outcome Measure” AND [“exp retinoblastoma” OR “exp Ophthalm” OR “exp Neoplasm”], in studies on populations aged 0–18 published in 2004 or later (the search terms “Patient-Reported Outcome” and “Patient-Reported Outcome Measure” were not indexed in MEDLINE or Embase prior to 2004, precluding the capture of relevant articles before this date). The search took place on February 10th, 2019 and was updated June 6th, 2020.

#### Eligibility criteria

Records in English that reported on PROMs used in retinoblastoma, pediatric oncology, or pediatric ophthalmology were included in the review. Grey literature, including conference abstracts and non-peer reviewed text, and records that reported on the preliminary phases of PROM development were excluded.

#### Study selection

The database searches identified study titles and abstracts which were screened for eligibility. All relevant full-text articles were assessed based on inclusion criteria by two independent reviewers (AJ, SL). Disagreements were resolved by a tie-breaker decision from a third study author (HD). Included full-text articles were read by study authors (AJ, SL) to identify unique PROMs.

#### Descriptive analysis

All unique PROMs underwent descriptive analysis. First, PROMs were classified by the construct they measured, based on the information provided in the included full-text articles (the research team did not independently assign construct labels). PROMs measuring the same constructs were grouped and the domains assessed by these PROMs were identified and tabulated.

PROMs were also classified as generic or condition-specific; generic PROMs are those that can assess a wide variety of conditions, and condition-specific PROMs are as those that examine the specific outcomes of a particular condition or aspect of care [10].

### Secondary objective

To evaluate the methodologic quality of the identified PROMs through application of the COSMIN ‘Good Measurement Property’ checklist.

#### Search strategy

Each PROM identified in the primary objective was subject to a subsequent, individual search. This separate search was necessary, in order to identify articles that assess the measurement properties of the identified PROMs. Databases MEDLINE and Embase were searched using terms “Patient-Reported Outcome”, the specific name of the PROM, and “exp Validation”, all limited to ages 0–18. The searches were conducted between May 20th and June 10th, 2019.

To audit the search strategy, one study author (AJ) randomly selected 10% of PROMs and determined the number of articles assessing measurement properties associated with each PROM. Performing an audit on 10% of included PROMs was guided by recommendations in literature [28, 29]. An additional search was then performed in MEDLINE and Embase, using an alternative search strategy recommended by COSMIN and created with the help of an information scientist. New search terms included “Patient-Reported Outcome” AND “Patient-Reported Outcome Measure” AND [“exp Retinoblastoma” or “exp Ophthalm” or “exp Neoplasm”] AND “specific PROM name” AND [“exp Validation” OR “exp Psychometrics”] for populations aged 0–18. The articles identified by the alternative search strategy were compared with the articles originally identified.

#### Eligibility criteria and study selection

Studies that reported on the quantitative evaluation of measurement properties for any of the previously identified PROMs were included and passed on to quality assessment.

#### Methodologic quality assessment

The COSMIN ‘Good Measurement Property’ checklist was used to determine the methodological quality of each PROM [30]. The ‘Good Measurement Property’ checklist assesses PROM quality across 10 measurement properties, distributed among three categories: (i) ‘overall content validity’ with subcategories ‘PROM development’ and ‘content validity’; (ii) ‘internal structure’ with subcategories ‘structural validity’ and ‘internal consistency’; and (iii) ‘remaining measurement properties’ with subcategories ‘reliability’, ‘measurement error’, ‘construct validity’, ‘criterion error’, ‘cross-cultural validity’, and ‘responsiveness’. COSMIN guidelines allow authors to determine which of the 6 ‘remaining measurement properties’ to include in their review; ‘reliability’, ‘measurement error’, and ‘construct validity’ were chosen for this study. The definition and judgment criteria for each measurement property followed COSMIN guidelines, as previously published [31].

All identified studies evaluating a given PROM’s measurement properties were read in full and measurement properties were identified. Sufficiency of each measurement property was judged against established criteria [31] and given a score of ‘+’, ‘-’, or ‘?’; if an individual measurement property fulfilled the established criteria it received ‘+’ indicating sufficient quality, if the property was not reported on it received ‘?’, and if the property did not fulfill the established criteria, it received ‘-’ to indicate insufficient quality. If identified studies reported discordant measurement property values for an instrument, the review team used the lower-score, i.e., ‘?’ is higher than ‘-’, consistent with COSMIN recommendations [31]. Two reviewers (AJ, SB) independently applied the good measurement property checklist across all identified studies evaluating measurement properties, for all PROMs. Discrepancies were resolved by a third reviewer (HD).

Finally, all PROMs, stratified by construct, were ranked based on the quality of individual measurement properties; a ‘+’ value was determined to be the highest value and ‘-’ the lowest value. Within a construct group, PROMs that received the most ‘+’ values across 7 individual properties were ranked highest.

## Results

### Primary objective

#### Study selection

**Fig. 1:**
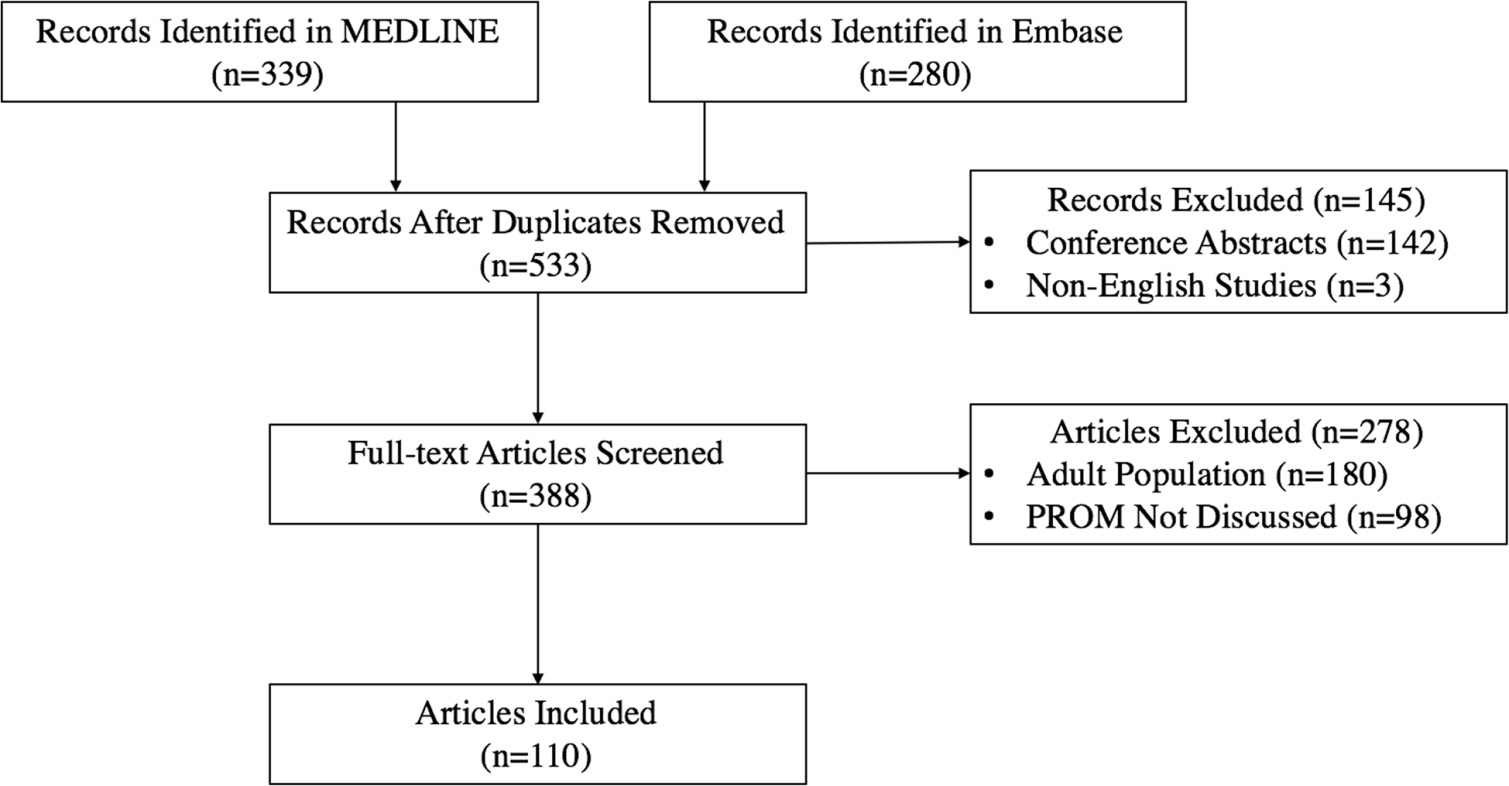
Process of study selection, consort diagram. Unique articles identified by comprehensive database searches (*n* = 533) were screened by title and abstract against inclusion and exclusion criteria. Full text articles (*n* = 388) were then reviewed against inclusion and exclusion criteria. Full-text review resulted in 110 included articles

**Fig. 2:**
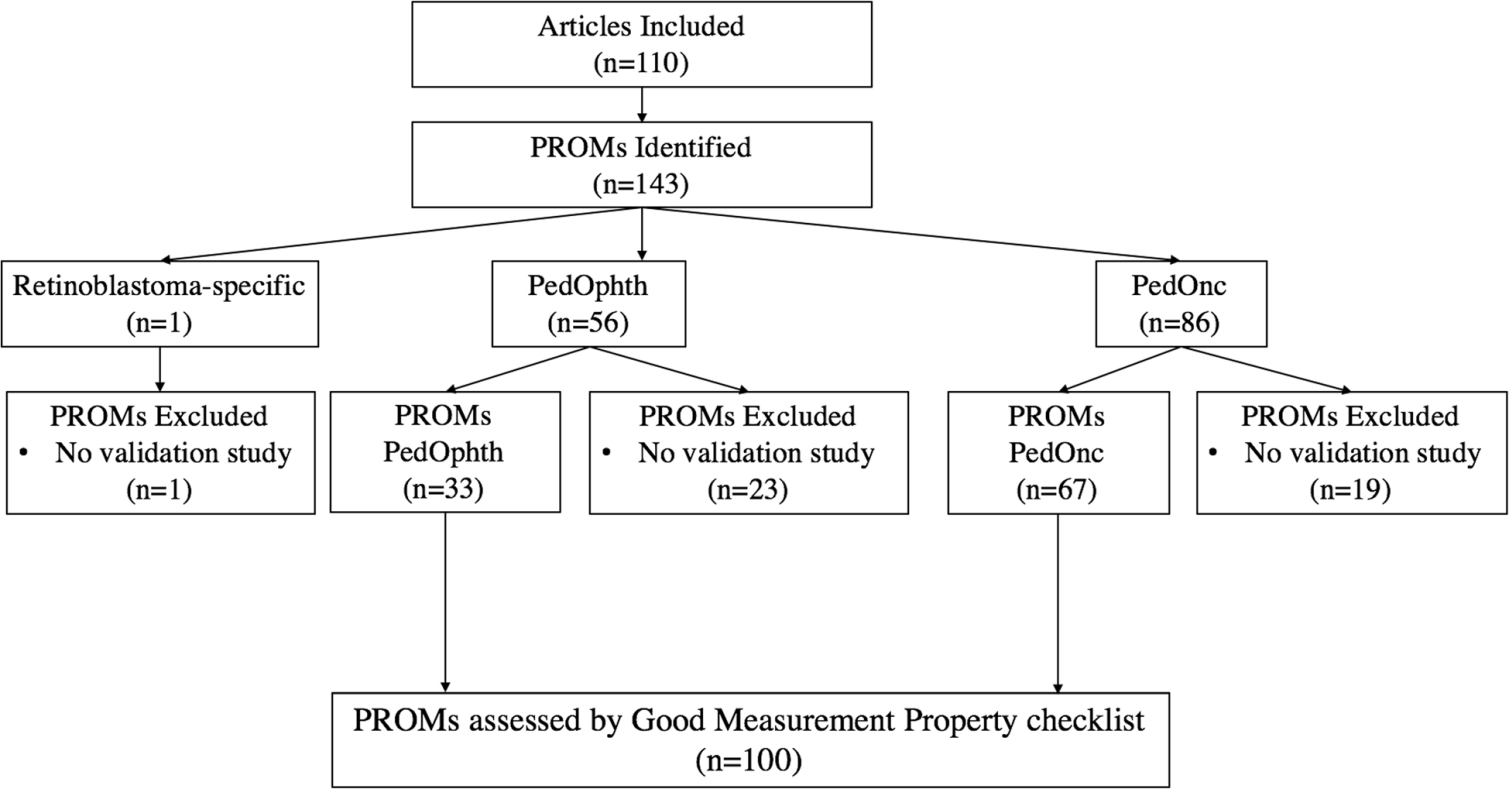
PROM identification and breakdown, flow diagram. Of the PROMs identified for use in the populations of interest (*n* = 143), validation articles (*n* = 100) and were subsequently were identified for pediatric ophthalmology (PedOphth) and pediatric oncology (PedOnc) PROMs. PROMs were then assessed by the COSMIN good measurement property checklist for methodologic quality

The MEDLINE and Embase search identified 533 unique manuscripts and after initial screening 145 were excluded (142 were grey literature and three were non-English studies). Full-text review of the remaining 388 articles yielded 110 manuscripts that met inclusion criteria (Fig. 1). These articles identified 143 unique PROMs: one used for retinoblastoma, 56 used in pediatric ophthalmology broadly, and 86 used in pediatric oncology broadly (Fig. 2). The inter-rater agreement between reviewers on screening and full-text review was 83% (444/533 manuscript agreement), adhering to standards in literature [32, 33].

#### Descriptive analysis

##### Constructs and domains

**Fig. 3:**
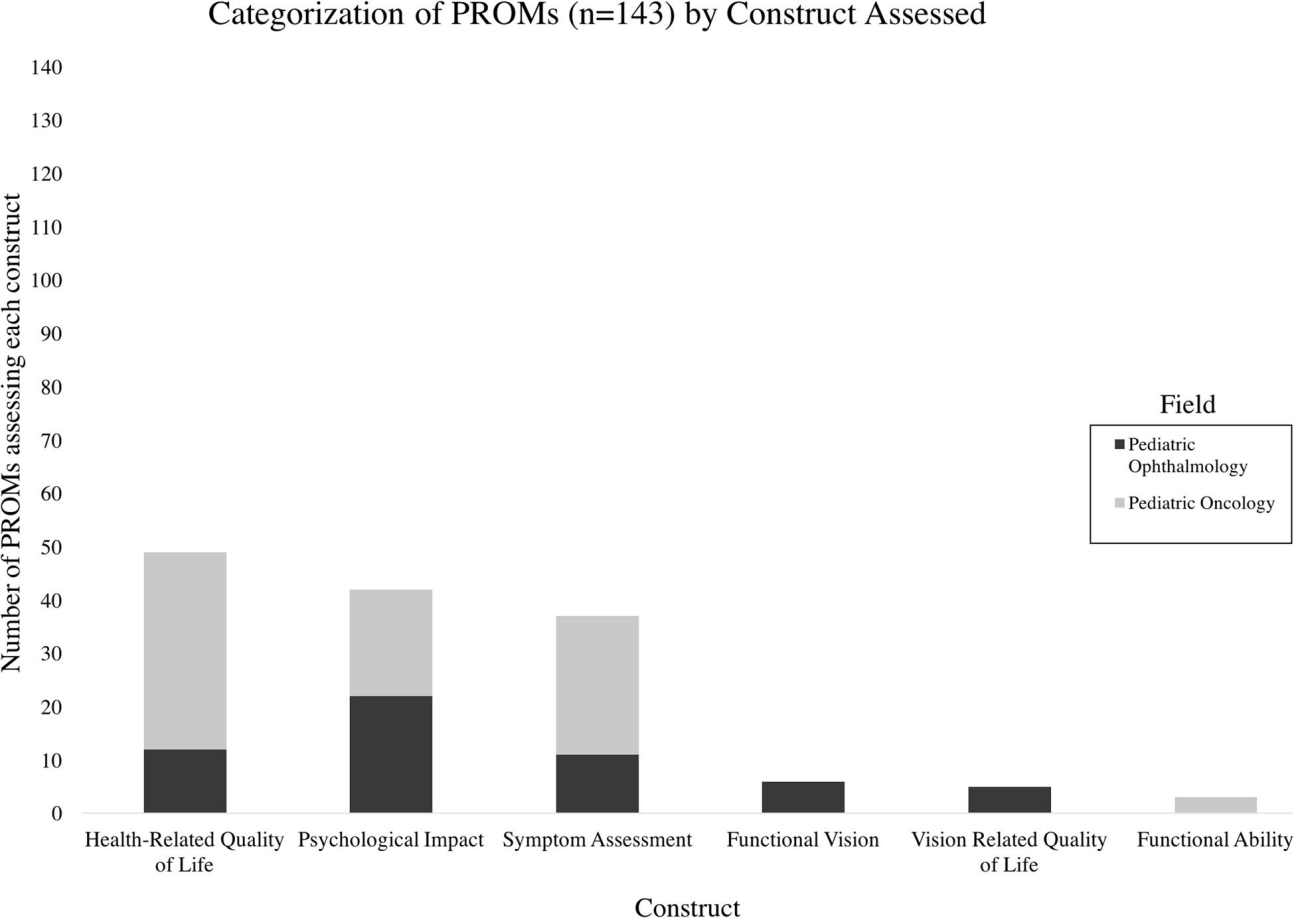
Categorization of PROMs by Construct Assessed. The PROMs identified in the scoping review (n = 143) were characterized by construct. Constructs, shown left to right, are ordered most frequently assessed to least frequently assessed. The most frequently assessed domains was ‘HRQoL’ (49/143) followed by ‘Psychological Impact’ (42/143), and ‘Symptom Assessment’ (37/143)

Descriptive analysis revealed the 6 constructs measured by 143 PROMs included: ‘Health-Related Quality of Life’ (HRQoL) (35%, 50/143), ‘Psychological Impact’ (29%, 42/143), ‘Symptom Assessment’ (22%, 37/143), ‘Functional Vision’ (FV) (4%, 6/143), ‘Vision-Related Quality of Life’ (VRQoL) (4%, 5/143), and ‘Functional Ability’ (2%, 3/143) (Fig. 3). One retinoblastoma-specific PROM was identified, measuring ‘HRQoL’. The majority of PROMs used in pediatric oncology measured ‘HRQoL’ (43%, 37/86), followed by ‘Symptom Assessment’ (30%, 26/86), ‘Psychological Impact’ (23%, 20/86), and ‘Functional Ability’ (4%, 3/86). Of PROMs used in pediatric ophthalmology, the majority measured ‘Psychological Impact’, (39%, 22/56) followed by ‘HRQoL’ (21%, 12/56), ‘Symptom Assessment’ (20%, 11/56). ‘FV’ (11%, 6/56), and ‘VRQoL’ (9%, 5/56).

**Fig. 4:**
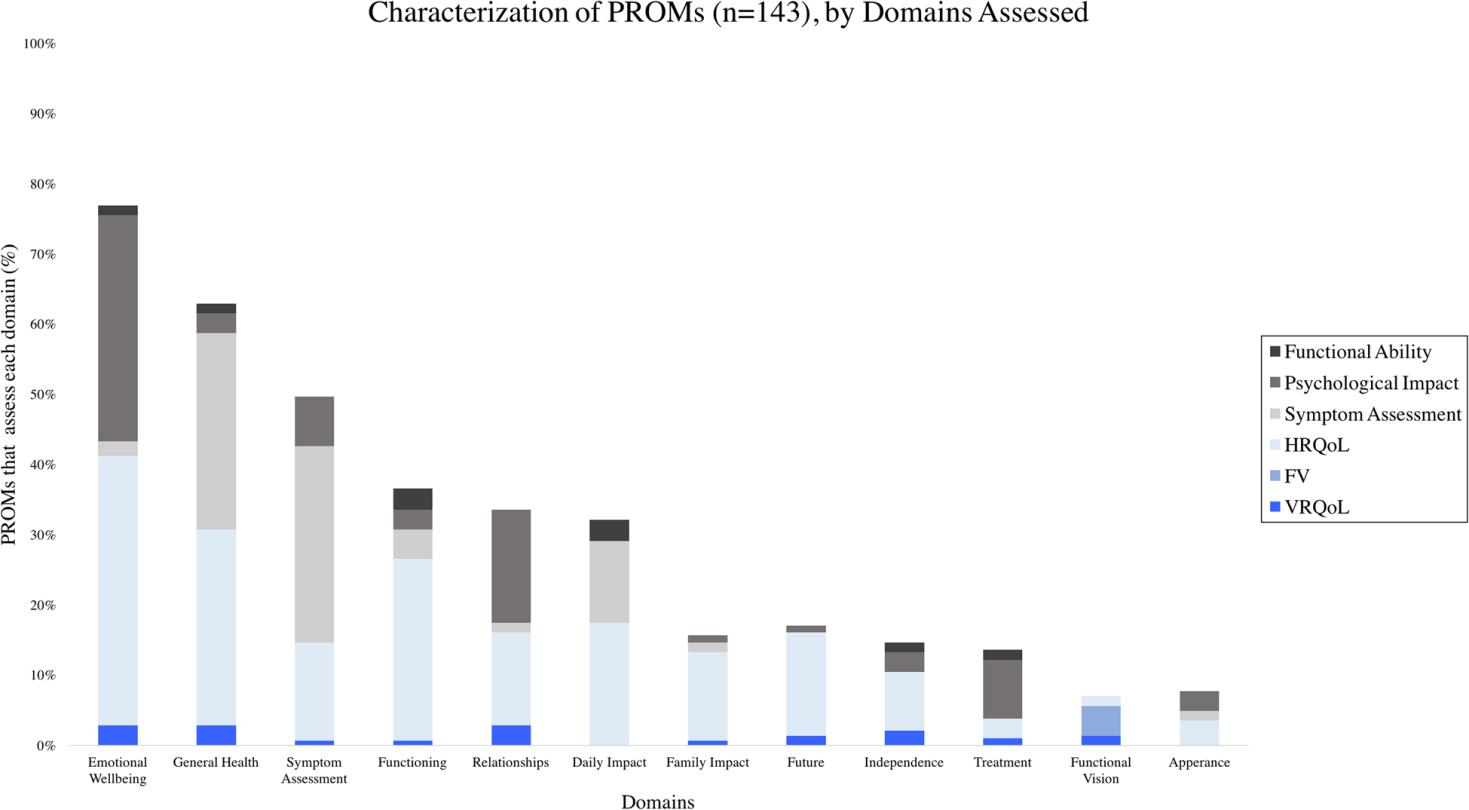
Characterization of PROMs by Domain Assessed. The domains assessed by each PROM (n = 143) were identified and their frequency tabulated. Domains, shown left to right, are ordered most frequently to least frequently assessed, stratified by construct. The most frequently assessed domain was emotional well-being (110/143), followed by general health (90/143), and symptom assessment (71/143)

Identification and tabulation of domains indicated emotional wellbeing to be assessed most often (80%, 110/143), followed by general health (63%, 90/143), symptom assessment (50%, 71/143), functioning (35%, 51/143), relationships (34%, 48/143), daily impact (23%, 33,143), family impact (17%, 25/143), future (16%, 23/143), independence (15%, 22/143), treatment (13%, 19/143), appearance (8%, 11/143) and FV (7%, 10/143) (Fig. 4).

##### Target population

Of the 143 PROMs, 61 (43%) were condition-specific measures and 82 (57%) were generic measures (AdditionalDataTable). Condition-specific measures were designed for retinoblastoma (1/61, 1%), other eye conditions (27/61, 44%), other cancers (18/61, 29%), and miscellaneous conditions (16/61, 26%). Of the PROMs assessing eye conditions, 13/27 (48%) assessed strabismus, 9/27 (33%) assessed amblyopia, and 5/27 (19%) assessed others (i.e. rhinoconjectivitis, cataracts, nasolacrimal pathway obstruction, exotropia, and vernal keratoconjunctivitis). Of PROMs assessing cancers, 16/18 (89%) were used for non-specific invasive malignancies such as leukemia or lymphoma, while 2/18 (11%) were used for patients undergoing bone marrow transplant (AdditionalDataTable).

### Secondary objective

#### Study selection

Of the 143 identified PROMs, the secondary MEDLINE and Embase searches identified articles assessing measurement properties for 100 PROMs – 33 used in pediatric ophthalmology and 67 used in pediatric oncology (Fig. 2). Notably, the retinoblastoma-specific PROM did not have any associated studies that assessed any measurement properties. A total of 191 studies (mean 1.91, median 2) were identified from database searches for the 100 PROMs.

The audit performed on 10% of PROMs revealed 95% agreement between the two search strategies, identifying 2 additional studies assessing the measurement properties of 2 PROMs; 33 studies were identified by through the initial method and 35 identified through the subsequent search. The two newly identified studies underwent COSMIN quality analysis and the results did not impact the original quality score.

#### Methodologic quality assessment results

The quality score of all PROMs that had associated measurement property assessment studies in both pediatric ophthalmology and oncology, stratified by construct, is summarized in Table 1. Analysis using the COSMIN ‘Good Measurement Property’ checklist revealed 34/100 PROMs received a score of sufficient quality in both subcategories of ‘overall content validity’: 15 used in pediatric ophthalmology and 19 used in pediatric oncology. Additionally, 3/100 received a score of sufficient quality in both subcategories of ‘internal structure’: one used in pediatric ophthalmology and two used in pediatric oncology. No identified PROM received a score of sufficient quality in all three subcategories of ‘remaining measurement properties’, however, 19/100 PROMs received a score of sufficient quality in two of its three subcategories, reliability and construct validity.

**Table 1:** Quality analysis of all identified PROMs (*n* = 100). The COSMIN ‘good property checklist’ was applied on 100 PROMs, across 7 measurement properties stratified by construct. PROMs were ranked highest score of sufficient quality to lowest, within a construct. ‘+’ indicates a score of sufficient quality, ‘?’ indicates that the specific measurement property was not reported on, and ‘-’ indicates insufficient quality

PROM	COSMIN Good Measurement Properties Checklist						
	Overall Content Validity		Internal Structure		Remaining Measurement Properties		
	PROM Development	Content Validity	Structural Validity	Internal Consistency	Reliability	Measurement Error	Hypotheses testing for construct validity
VRQoL							
Vision Related Quality of Life (VQoL_CYP)	+	+	?	+	–	?	+
Impact of Vision Impairment for Children (IVI_C)	+	+	?	+	–	?	+
National Eye Institute-Visual Function Questionnaire (NEI-VFQ)	?	+	?	+	+	?	+
Effects of Youngsters’ Eyesight on Quality of Life (EYE-Q)	+	–	?	+	+	?	+
Childrens Visual Function Questionnaire (CVFQ)	–	–	?	–	–	?	?
FV							
Functional Vision Quality of Life (FVQ_CYP)	+	+	?	+	?	?	+
Cardiff Visual Ability Questionnaire for Children (CVAQC)	+	+	–	+	+	?	?
LV Prasad–Functional Vision Questionnaire (LVP-FVQ II)	+	+	?	?	+	?	?
Visual Analogue Scale	?	?	?	?	+	?	?
Visual Function Index-14	–	–	?	+	?	?	?
HRQoL							
Patient Reported Outcome Measure Information System (PROMIS) Pediatric Profile-25	+	+	+	+	?	?	+
Pediatric Rhinoconjunctivitis Quality of Life Questionnaire (PRQLQ)	+	+	?	+	+	?	+
Early-Onset Scoliosis Questionnaire (EOSQ)	+	+	?	+	+	?	+
Adult Strabismus-20 (AS-20)	+	+	?	+	+	?	?
Amblyopia and Strabismus Questionnaire (A&SQ)	+	+	?	+	+	?	?
Bt-Dux	+	+	?	+	?	?	+
Minneapolis–Manchester Quality of Life (MMQL) Questionnaire	+	+	?	+	–	?	+
KIDSCREEN 52	+	+	?	+	–	?	+
Child Health Ratings Inventories (CHRIs - HSCT)	+	+	?	+	–	?	+
Lee Chronic Graft Versus Host Disease (Lee-cGVHD)	+	+	?	+	–	?	+
Child Health and Illness Profle (CHIP)	+	+	?	+	–	?	+
Quality of Life in Children with Vernal Keratoconjunctivitis (QUICK)	+	+	?	+	–	?	+
Intermittent Exotropia Questionnaire (ITXQ)	+	+	–	+	+	?	?
EuroQol 5-Dimension Youth (EQ-5D-Y)	+	+	?	?	+	?	+
Royal Marsden Hospital Pediatric Oncology Quality of Life Questionnaire (RMH-PQLQ)	+	+	?	+	?	?	?
Nasolacrimal Duct Obstruction (NLDO) Questionnaire	+	+	?	+	?	?	?
The Cancer Needs Questionnaire-Young People (CNQYP)	+	+	?	+	–	?	?
Quality of Life for Children with Cancer (QOLCC)	+	+	–	+	?	?	?
Children’s Amblyopia Treatment Quality of Life Questionnaire (CAT-QoL)	+	+	–	+	?	?	–
Health Utilities Index (HUI)	+	+	?	?	?	?	+
Coddington Life Events Questionnaire (CLES)	+	+	?	?	–	?	+
Perceived Illness Experience (PIES)	+	+	?	–	?	?	+
TNO-AZL Preschool Children Quality of Life (TAPQOL)	+	+	?	–	–	?	+
Pediatric Quality of Life Inventory—Cancer Module (PedsQL Cancer)	+	?	?	+	+	?	?
MPQOLQ Miami Pediatric Quality of Life Questionnaire	+	?	?	+	+	?	?
M.D. Anderson Symptom Inventory	–	–	?	+	+	?	+
PainDETECT	+	–	?	+	?	?	?
World Health Organisation Quality of Life assessment (WHOQoL) BREF	–	+	–	+	?	?	+
The Hope Scale	–	+	?	+	?	?	?
The Adolescent Quality of Life Questionnaire	–	+	?	?	?	?	+
KINDL	?	?	?	+	+	?	?
Medical Outcomes Study Short-Form 36 (SF-36)	–	–	?	+	?	?	+
Infant Toddler Quality of Life Inventory (ITQOL)	–	–	?	+	?	?	+
Functional Assessment of Cancer Therapy - General (FACT - G)	?	?	?	+	?	?	+
16-Dimensional Health-related Measure (16D)	–	+	?	?	?	?	?
Child Health Questionnaire (CHQ)	?	?	?	+	?	?	?
European Organization for Research and Treatment of Cancer Quality of Life Questionnaire (EORTC QLQ-C30)	?	?	–	?	?	?	+
Pediatrics Outcomes Data Collection Instrument (PODCI)	?	?	–	?	?	?	?
Behavioral, Affective, and Somatic Experiences Scale (BASES)	–	–	?	+	?	?	?
Scoliosis Research Society [SRS]-30	?	?	?	–	?	?	?
Pediatric Quality of Life (PEDQOL)	–	–	?	–	?	?	?
Functional Ability							
Toronto Extremity Salvage Score (TESS)	+	+	?	+	+	?	+
DISABKIDS	+	+	?	+	?	?	+
The Activities Scale for Kids Performance Version (ASKp)	+	–	?	?	+	?	+
Symptom Assessment							
Amblyopia Treatment Index (ATI)	+	+	?	+	+	?	?
Chronic Otitis Media Questionnaire-12 (COMQ-12)	–	+	+	+	–	?	+
Oral Health Impact Profile (OHIP-14)	–	+	?	+	–	?	+
Hipdisability Osteoarthritis Outcome Score (HOOS)	+	–	?	+	+	?	+
The Cancer and Treatment Distress (CTXD)	–	+	?	+	?	?	?
Children’s Vision for Living Scale (CVLS)	+	+	–	+	?	?	?
Oral Mucositis Daily Questionnaire (OMDQ)	–	+	?	–	?	?	+
Brief Fatigue Inventory	?	?	?	+	+	?	+
Impact of Events Scale-Revised (IES-R)	?	?	–	+	+	?	+
Memorial Symptom Assessment Scale	–	+	?	+	?	?	+
Fatigue Symptom Inventory	–	+	?	+	?	?	+
Adolescent Pediatric Pain Tool (APPT)	+	–	?	+	?	?	+
Knee-injury osteoarthritis outcome score (KOOS)	+	–	?	?	+	?	+
Hearing Measurement Scale	–	+	?	?	?	?	?
Short Form McGill Pain Questionnaire	–	+	–	+	?	?	?
Expectations of Strabismus Surgery Questionnaire	–	+	?	?	?	?	?
Kessler-6 Two Factor	–	–	?	+	?	?	+
Colored Analogue Scale	–	–	?	?	+	?	+
Edmonton Symptom Assessment Scale (ESAS)	?	?	?	+	?	?	?
Wong-Baker FACES Pain Rating Scale	?	?	?	?	?	?	+
Symptom Checklist 90 R	–	–	?	?	?	?	+
Brief Pain Inventory Short Form	–	–	?	?	?	?	?
Emotional Impact of Amblyopia Questionnaire (EIAQ)	–	–	?	?	?	?	?
Psychological Impact							
Derriford Appearance Scale (DAS)	+	+	?	+	+	?	+
Multidimensional Scale of Perceived Social Support (MSPSS)	?	+	?	+	+	?	+
Hospital Anxiety and Depression Scale (HADS)	–	+	+	+	?	?	+
Screen for Child Anxiety Related Emotional Disorders (SCARED)	–	+	?	+	+	?	+
Zung Self-Rating Depression Scale	+	?	?	+	?	?	+
Beck Anxiety Inventory	–	+	–	+	?	?	+
Hopkins Symptom Check List (HSCL)	–	–	?	+	+	?	+
Fear of Negative Evaluation (FNE)	–	–	?	+	+	?	+
Liebowitz Social Anxiety Scale	–	–	–	+	+	?	+
Psychological Impact Questionnaire (PIQ)	+	+	?	?	?	?	?
Childrens Manifest Anxiety Scale (CMAS)	–	+	?	+	?	?	?
Hamilton Anxiety Rating Scale	?	?	–	+	?	?	+
Distressed Personality Questionnaire (DS-14)	?	?	?	+	–	?	+
Self-Perception Profile for Children (SPPC)	?	?	–	+	–	?	+
Behavioural Assessment System for Children (BAS-C)	–	–	+	+	?	?	–
Social Anxiety Scale for Children-Revised	–	–	?	+	?	?	+
Mental Health Inventory (MHI)	–	–	?	+	?	?	+
Center for Epidemiologic Studies Depression Scale – Revised (CESD-R)	–	–	–	+	?	?	+
Perceived Psychosocial Questionnaire (PPQ)	+	–	?	?	?	?	?
Children’s Depression Inventory (CDI)	?	?	?	+	–	?	–
State-Trait Anxiety Inventory	–	–	?	+	–	?	?
Pediatric Camp Outcome Measure (PCOM)	–	–	?	+	?	?	?
The Psychosocial Assessment Tool (PAT)	–	–	?	–	?	?	+
Faces Anxiety Scale	–	–	?	?	?	?	?

The Patient-Reported Outcome Measure Information System (PROMIS) Pediatric Profile-25 [34–36], a measure commonly used in pediatric oncology, was the only PROM to receive a score of sufficient quality in both ‘overall content validity’ and ‘internal structure’. The PROMIS Pediatric Profile-25 was also one of only 5 PROMs used in pediatric oncology to receive a score of sufficient quality in 5 of 7 measurement property subcategories (Table 1). The other PROMs meeting this score included; two ‘HRQoL’ measures: Pediatric Rhinoconjunctivitis Quality of Life Questionnaire (PRQLQ) [37, 38] and the Early-onset Scoliosis Questionnaire (EOSQ) [39–41], one ‘Functional Ability’ measure: Toronto Extremity Salvage Score (TESS) [42], and one ‘Psychological Impact’ measure: the Derriford Appearance Scale (DAS) [43, 44] (Table 1). Additionally, all of these PROMs had good ‘overall content validity’; however, unlike PROMIS Pediatric Profile-25, they did not score well on internal structure.

The 6 highest-scoring PROMs used in pediatric ophthalmology each received a score of sufficient quality in four of 7 measurement property subcategories (Table 1). These included four ‘VRQoL’ measures: National Eye Institute-Visual Function Questionnaire (NEI-VFQ) [45, 46], Effects of Youngsters’ Eyesight on Quality of Life (EYE-Q) [47, 48], Vision-Related Quality of Life for Children and Young People (VQoL_CYP) [49, 50], Impact of Vision Impairment for Children (IVI_C) [51, 52] and two measures of ‘Functional Vision’: Functional Vision Quality of Life for Children and Young People (FVQ_CYP) [53] and Cardiff Visual Ability Questionnaire for Children (CVAQC) [54]. Of these, 4/6 (i.e. VQoL_CYP, IVI_C, FVQ_CYP and CVAQC) received a score of sufficient quality in ‘overall content validity’.

The inter-rater percent agreement and Kappa coefficient between quality scores from independent reviewers across all measurement properties were 92% and 0.79, respectively; both values adhere to standards in literature [32, 33, 55]. The percent agreement and Kappa coefficients for individual categories was as follows: PROM development (91%, 0.82), content validity (90%, 0.79), structural validity (92%, 0.65), internal consistency (95%, 0.87), reliability (78%, 0.65), measurement error (96%, 0.78), and construct validity (85%, 0.98). There were no discordant measurement property values reported between studies analyzing the same PROM.

## Discussion

### General discussion

This review identified 143 PROMs that have been used to assess outcomes in retinoblastoma, pediatric ophthalmology, and pediatric oncology populations. One retinoblastoma-specific PROM was identified, however, it was not built with help of patients and its methodologic quality has yet to be assessed [56]. Several PROMs with variable validity and reliability were identified that are currently used in pediatric ophthalmology and oncology. No PROMs received a perfect quality score indicating that all could benefit from further psychometric improvements, such as improving their content validity, increasing reliability, or defining measurement error associated with use.

Patient participation in the development of PROMs is of paramount importance and is becoming more commonplace [30, 57–59]. Conversations with patients can help highlight important domains of the construct to be assessed, which is essential for strong content validity, i.e. the degree to which the content of an instrument reflects the underlying construct [13, 60]. The results of this research indicated that of all PROMs that received a sufficient score in ‘overall content validity, over two-thirds measured ‘HRQoL’. In contrast, few PROMs measuring ‘Symptom Assessment’ and ‘Psychological Impact’ received a sufficient score in ‘overall content validity’; the vast majority of these PROMs did not meet COSMIN criteria in either subcategory of ‘overall content validity’, implying insufficient patient involvement in their development. Possible explanations for low patient involvement during the development of the ‘Symptom Assessment’ construct have been suggested previously. For example, patient input might be deemed unnecessary when the specific condition in question is well documented clinically, with the presumption that no new information would be gleaned from patient participation [61]. More broadly in PROM development, absence of patient involvement could be due to time and budget constraints that researchers face [61] when developing a PROM; the financial costs of patient involvement, frequently recognized in literature as a challenge of qualitative research broadly [62], can contribute to a researcher team’s decision to not involve patients. However, despite these challenges, researchers have started to shift their thinking and recognize the positive implications of patient participation and the value of patient experience [11, 24, 27, 30]. Additionally, several funding bodies and regulation bodies, such as the Canadian Institute for Health Research and the US Food and Drug Administration have made patient involvement in PROM development mandatory [63, 64].

The review identified no PROMs that received a sufficient quality score across all three ‘remaining measurement properties’ (i.e. reliability, construct validity, and measurement error). One explanation for this could be due to the strict statistical criteria outlined by the ‘Good Measurement Property’ checklist. The checklist uses well-established means for evaluating measurement properties [65], yet it provides a very narrow list of options for evaluation, often only accepting specific statistical tests. For instance, in practice, an acceptable evaluation of a PROM’s reliability can include determining the interclass coefficient, weighted kappa, or Pearson’s r Coefficient, yet the COSMIN ‘Good Measurement Property’ checklist only acknowledges the former two as acceptable values [30]. Therefore, PROMs demonstrating strong reliability solely through Pearson’s r Coefficient would not meet COSMIN criteria. Additionally, the definition of reliability involves examining internal consistency, reliability, and measurement error; however, we found that while internal consistency and reliability were commonly reported, measurement error was often omitted. Beyond COSMIN, the recommendations regarding reporting of measuring property provides mixed direction; some experts recommend assessment and reporting of all measurement properties of broad reliability and validity [66], while others argue that related measurement properties should not be assessed or reported as separate dimension [67]. Thus, it remains unclear if the oft-omitted measurement error in the studies we assessed was actually assessed but not reported (to avoid redundancy), or if it was not assessed at all.

As the name implies, generic PROMs are intended for use on any disease population to allow for comparability across patient populations [10]. However, since generic measures are not designed to capture areas of concern to specific patient populations [12, 22, 68], they are likely to include irrelevant questions for certain patient groups or omit issues that are specific to a particular condition [68–70]. Use of generic measures to assess specific conditions may result in i) capturing data that are not meaningful to patients [22, 68], ii) failure to accurately measure what the PROM purports to measure [57, 58, 68], and iii) producing inaccurate comparisons between groups [68–70]. Where the content validity of generic PROMs may be questionable, condition-specific PROMs are preferred [71]. Literature regarding development of condition-specific PROMs recommends patient co-development [12, 22, 57, 68], i.e. developing questions by means of qualitative methods with relevant patients and thoroughly testing the measurement properties of novel PROMs with new populations of patients [71, 72]. This results in PROMs that address aspects of an outcome that are important for a particular patient population, facilitating good validity and responsiveness of the measure. Yet, despite the described importance of patient co-development, our study identified several condition-specific self-report outcome measures which had poor PROM development and content validity. In particular, patients were not included in the development of many of these condition-specific PROMs (Table 1), therefore they may be lacking a focus on topics or questions meaningful to the patient experience.

Patient co-development of PROMs is likewise encouraged by theoretical frameworks used to guide construct development, as has been robustly demonstrated in development of several PROMs assessing ‘HRQoL’ [72, 73]. When there is an absence of clarity surrounding the initial definition of a construct, this can lead to: development of measures that provide inaccurate inferences about outcomes; difficulty deciding on what domains to include in a measure; and challenges in comparing, generalizing, and replicating research findings. Examples of such confusion include inconsistent definitions of ‘HRQoL’ used by several PROMs [74, 75], and conflation of related, but distinct, constructs, such as ‘VRQoL’ and ‘FV’ [24, 58, 76]. Prior research on pediatric oncology PROMs highlighted measures, of which several are assessed in this study, that were developed without precisely conceptualizing and defining the construct ‘HRQoL’ [75, 77]. One such measure is the Pediatric Quality of Life Inventory – Cancer Module (PedsQL - Cancer) [77]; this is consistent with our study, in which there was insufficient published data to allow evaluation of content and construct validity (Table 1). Perhaps then, informing the construct definition through insights gleaned from qualitative conceptual discussions with patients, parents, researchers, and health-care professionals, may improve several psychometric measurement properties [73, 78].

Looking towards the development of a retinoblastoma-specific PROM, care must be taken to ensure that the constructs and domains measured are relevant and valuable to the patient population. Some research groups have argued that ‘HRQoL’ is the most important construct to assess for retinoblastoma [7, 56], however the patient voice is largely missing from the literature. RetinoQuest [56], the retinoblastoma-specific PROM identified by our study, is an ‘HRQoL’ measure which has yet to be assessed for its methodologic quality. Furthermore, RetinoQuest lacks a conceptual definition of ‘HRQoL’ and retinoblastoma patients were not consulted during its development. A way forward for this measure could be to work alongside retinoblastoma patients to first determine if this is the appropriate construct to assess, then refine the definition and theoretical underpinning of ‘HRQoL’. Another approach could be to adapt an existing well-validated ‘HRQoL’ measure to the retinoblastoma context; for example, the PROMIS Pediatric Profile-25 proved strong methodological quality as assessed in our study, and it also demonstrates a well-defined theoretical underpinning of ‘HRQoL’ [75, 77].

Several research studies have indicated that ‘Psychological Impact’ maybe another important construct to consider for a retinoblastoma-specific PROM [3, 5, 9]. The DAS, a generic measure assessing the psychologic impact of appearance, scored highest in our study in the ‘Psychological Impact’ construct category [43, 44], receiving a score of sufficient quality for ‘overall content validity’. Since retinoblastoma patients and their families have voiced concerns regarding cosmetic appearance after treatment [3, 5], perhaps with appropriate adaptation and methodologic assessment, the DAS can be explored for use in the retinoblastoma population. Alternatively the Psychological Impact Questionnaire (PIQ) [79], a condition-specific amblyopia measure, could similarly be explored for relevance. The PIQ was the only other ‘Psychologic Impact’ PROM to receive a score of sufficient quality for ‘overall content validity’, but did not report on any of the other remaining measurement properties. A future step could be to utilize qualitative methods to determine if ‘Psychological Impact’ is truly an important construct to evaluate for retinoblastoma, and if it is, what facets of ‘Psychological Impact’ are valued by retinoblastoma patients.

### Strengths and limitations

This review has several important strengths. First, it was conducted with methodologic rigor, adhering to COSMIN PROM evaluation guidelines, and used two independent reviewers for study selection (83% inter-rater agreement) and quality analysis (92%, 0.79 inter-rater percent agreement and Kappa Coefficient), reliability that is comparable to recommendations in literature [32, 33]. Second, our review revealed that numerous PROMs for children suffering from various ophthalmic-conditions or cancer whose measurement properties have not been sufficiently assessed for use in these populations, several with significant gaps in their methodologic quality. In keeping with guidelines in literature for the development of adult PROMs [80], a self-report outcome measure should not be put into practice without careful consideration of its psychometric strengths and limitations. Yet, all of the PROMs identified by this review are in use today. Moving forward, researchers should consider assessing the methodologic quality of all these self-report outcome measures to follow standardized, international COSMIN guidelines before integrating the PROM into a clinical setting. This review has practical significance. Several generic and condition -specific PROMs with sufficient methodologic quality have been identified that could potentially be relevant for adaptation and application to retinoblastoma patients, with subsequent consultation with patients, experts, and pertinent literature. In this vein, to inform the development of a retinoblastoma-specific PROM, next steps could include qualitative discussions with retinoblastoma patients and their caregivers to uncover the treatment outcomes they find valuable and relevant to their care.

Our findings should be interpreted in light of several limitations. First, for practical reasons, we chose to only include English publications. However, as the initial search strategy identified three non-English manuscripts out of 533, it is reasonable to assume that there are unlikely to be many non-English studies relevant to this topic. Second, the authors acknowledge that more PROMs may have been identified if databases beyond just MEDLINE and Embase had been searched. However, our methods were consistent with search strategy recommendations of the COSMIN guidelines [30], and still yielded a relatively large number of relevant PROMs. Third, the initial keyword search, though developed with an information scientist, could have included more comprehensive search terms. Fourth, identification of validation articles for individual PROMs was performed via a separate search in MEDLINE and Embase, and was not included in the initial search strategy. The decision for the second search was necessary to reflect the changes made to the study protocol, the addition of a methodologic quality assessment. Therefore, this might account for articles discussing the analysis of some measurement properties that were not identified. However, in attempt to determine the accuracy of our search method, we audited 10% of the identified instruments and conducted a search following COSMIN systematic review guidelines [30]. A 10% audit is commonly applied in practice [32, 33]. The audit revealed only two new studies assessing measurement properties associated with two PROMs’, suggesting that a larger audit would be unlikely to identify more studies. In addition, after quality analysis was applied to these studies, the associated PROM score did not change, suggesting that a larger audit would be unlikely to change the scores even if additional studies were retrieved. Finally, this review was not registered prior to its initiation. Although registration is not commonly required for scoping reviews, it does increase transparency and reproducibility and adherence to preregistered protocol helps decrease reporting bias. However, the study protocol underwent review, by several study authors (AJ, JS, and HD), and no deviations to the methodology were made throughout the study.

## Conclusion

In summary, this review identified many PROMs with variable reliability and validity that are currently used in retinoblastoma and related fields. Twelve PROMs were identified with good methodological quality, and based on the constructs and domains they assess, their potential application to retinoblastoma has been considered. Only one retinoblastoma-specific PROM, that was not developed with the help of patients and has yet to be validated, was identified. Therefore, moving forward it is critical to develop, validate, and implement outcome measures for retinoblastoma patients. Future research could involve patients and experts to jointly examine patient’s perspectives and agree on the specific constructs to be measured by a future retinoblastoma PROM. Additionally, adapting the relevant high-scoring PROMs to include retinoblastoma-specific domains could be explored. This could be done through pursuing qualitative methods of patient participation to help identify which outcome domains are important to patients. Once constructs, domains, and the number of measures need have been identified, validating these measures on a retinoblastoma patient-population, in adherence to COSMIN guidelines, will be important in order to ensure thorough reliability and validity.

## Supplementary information

**Additional file 1: Additional Data Table.** Characterization of Condition-specific PROMs (*n* = 61). Of the condition-specific PROMs identified, majority (27/61) were designed for ophthalmic conditions, followed by cancer-specific measures (18/61), miscellaneous conditions (16/61), and retinoblastoma (1/61).

## Data Availability

The database records identified by the literature search and their characterization, as indicated in Figs. 1, 2, 3 and 4, are available from the corresponding author on reasonable request.

## Abbreviations

COSMIN: COnsensus-based standard for the Selection of health Measurement INstruments
FV: Functional Vision
HRQoL: Health-Related Quality of Life
PROM: Patient-Reported Outcome Measure
VRQoL: Vision-Related Quality of Life
