# Supplementary material for: Patient-reported outcome measures for retinoblastoma: a scoping review
Source: J Patient Rep Outcomes. 2020 Aug 8;4:66. doi: 10.1186/s41687-020-00232-7 (PMC7415058; doi:10.1186/s41687-020-00232-7)
Supplement: Supplementary file 1 — Additional file 1: Additional Data Table. Characterization of Condition-specific PROMs (n = 61). Of the condition-specific PROMs identified, majority (27/61) were designed for ophthalmic conditions, followed by cancer-specific measures (18/61), miscellaneous conditions (16/61), and retinoblastoma (1/61). [file 41687_2020_232_MOESM1_ESM.docx]

| **Patient-Reported Outcome Measure** | **Condition** |
| --- | --- |
| Children’s Amblyopia Treatment Quality of Life Questionnaire (CAT-QoL) | Amblyopia |
| Amblyopia Treatment Index (ATI) | Amblyopia |
| Psychological Impact Questionnaire (PIQ) | Amblyopia |
| Emotional Impact of Amblyopia Questionnaire (EIAQ) | Amblyopia |
| Amblyopia Survey | Amblyopia |
| Children's Vision for Living Scale (CVLS) | Amblyopia |
| 46‐item Amblyopia QoL Questionnaire | Amblyopia |
| QoL questionnaire for children with anisometropic amblyopia | Amblyopia |
| Amblyopia and Strabismus Questionnaire (A&SQ) | Amblyopia and Strabismus |
| Adult Strabismus‐20 (AS‐20) | Strabismus |
| [Perspectives Questionnaire](https://onlinelibrary-wiley-com.myaccess.library.utoronto.ca/doi/full/10.1111/cxo.12553?sid=Ovid%3Aembase#cxo12553-bib-0074) | Strabismus |
| Disability Questionnaire | Strabismus |
| [Repertory Grid](https://onlinelibrary-wiley-com.myaccess.library.utoronto.ca/doi/full/10.1111/cxo.12553?sid=Ovid%3Aembase#cxo12553-bib-0066) | Strabismus |
| Perceived Visibility of Strabismus | Strabismus |
| [Expectations of Strabismus Surgery Questionnaire](https://onlinelibrary-wiley-com.myaccess.library.utoronto.ca/doi/full/10.1111/cxo.12553?sid=Ovid%3Aembase#cxo12553-bib-0073) | Strabismus |
| [Psychosocial Experience Questionnaire](https://onlinelibrary-wiley-com.myaccess.library.utoronto.ca/doi/full/10.1111/cxo.12553?sid=Ovid%3Aembase#cxo12553-bib-0067) | Strabismus |
| [8‐item QoL instrument](https://onlinelibrary-wiley-com.myaccess.library.utoronto.ca/doi/full/10.1111/cxo.12553?sid=Ovid%3Aembase#cxo12553-bib-0064) | Strabismus |
| [Exotropia Symptom Questionnaire](https://onlinelibrary-wiley-com.myaccess.library.utoronto.ca/doi/full/10.1111/cxo.12553?sid=Ovid%3Aembase#cxo12553-bib-0069) | Strabismus |
| [Effect of Diplopia Questionnaire](https://onlinelibrary-wiley-com.myaccess.library.utoronto.ca/doi/full/10.1111/cxo.12553?sid=Ovid%3Aembase#cxo12553-bib-0076) | Strabismus |
| [Post Strabismus Surgery Symptom Questionnaire](https://onlinelibrary-wiley-com.myaccess.library.utoronto.ca/doi/full/10.1111/cxo.12553?sid=Ovid%3Aembase#cxo12553-bib-0075) | Strabismus |
| Psychosocial effects of strabismus pre‐ and post‐operative questionnaire | Strabismus |
| [Satisfaction of Surgical Outcome](https://onlinelibrary-wiley-com.myaccess.library.utoronto.ca/doi/full/10.1111/cxo.12553?sid=Ovid%3Aembase#cxo12553-bib-0070) | Strabismus |
| Pediatric Rhinoconjunctivitis Quality of Life Questionnaire (PRQLQ) | Rhinoconjunctivitis |
| Quality of Life in Children with Vernal Keratoconjunctivitis (QUICK) | Vernal Keratoconjunctivitis |
| Intermittent Exotropia Questionnaire (ITXQ) | Exotropia |
| Retinoquest | Retinoblastoma |
| Visual Function Index-14 | Cataracts |
| Nasolacrimal Duct Obstruction Symptom Score (NLDO-SS) Questionnaire | Nasolacrimal Pathway Obstruction |
| Pediatric Quality of Life Inventory—Cancer Module (PedsQL Cancer) | Cancer, non-specific |
| Functional Assessment of Cancer Therapy - General (FACT - G) | Cancer, non-specific |
| Pediatric Quality of Life (PEDQOL) | Cancer, non-specific |
| Pediatric Patient-Reported Outcomes version of the Common Terminology Criteria for Adverse Events (PRO-CTCAE) | Cancer, non-specific |
| Quality of Life for Children with Cancer (QOLCC) | Cancer, non-specific |
| Minneapolis–Manchester Quality of Life (MMQL) Questionnaire | Cancer, non-specific |
| MPQOLQ Miami Pediatric Quality of Life Questionnaire | Cancer, non-specific |
| The Adolescent Quality of Life Questionnaire | Cancer, non-specific |
| RMH-PQLQ Royal Marsden Hospital Pediatric Oncology Quality of Life Questionnaire | Cancer, non-specific |
| European Organization for Research and Treatment of Cancer Quality of Life Questionnaire (EORTC QLQ-C30) | Cancer, non-specific |
| Brief Pain Inventory Short Form | Cancer, non-specific |
| Wong-Baker FACES Pain Rating Scale | Cancer, non-specific |
| Pediatrics Outcomes Data Collection Instrument (PODCI) | Cancer, non-specific |
| Adolecsent PRO-CTCAE | Cancer, non-specific |
| Measure yourself concerns and wellbeing (MYCaW) | Cancer, non-specific |
| M.D. Anderson Symptom Inventory | Cancer, non-specific |
| Bt-Dux | Cancer, non-specific |
| Behavioral, Affective, and Somatic Experiences Scale (BASES-C) | Bone Marrow Transplant |
| Toronto Extremity Salvage Score (TESS) | Musculoskeletal Injury |
| Oral Health Impact Profile (OHIP-14) | Decayed Teeth |
| Lee Chronic Graft Versus Host Disease (Lee-cGVHD) | Chronic Graft Versus Host Disease |
| Hopefulness Scale for Adolescents (HSA) | Bone Marrow Transplant |
| The Activities Scale for Kids Performance Version (ASKp) | Unilateral upper extremity deficiency |
| Hearing Measurement Scale | Cochlear Disability |
| Tinnitus Questionnaire for Auditory Brainstem Implant | Tinnitus |
| Knee-injury osteoarthritis outcome score (KOOS) | Knee Injury |
| Hipdisability osteoarthritis outcome score (HOOS) | Hip Injury |
| WHO Oral-Toxicity scale | Oral Mucositis |
| Oral Mucositis Daily Questionnaire (OMDQ) | Oral Mucositis |
| Early - Onset Scoliosis Questionnaire (EOSQ) | Scoliosis |
| Scoliosis Research Society (SRS)-30 | Scoliosis |
| Hand20 | Upper Limb Disorder |
| Pediatric Camp Outcome Measure (PCOM) | Cardiac Abnormalities |
